# IL-2 Signaling Axis Defects: How Many Faces?

**DOI:** 10.3389/fped.2021.669298

**Published:** 2021-07-02

**Authors:** Filippo Consonni, Claudio Favre, Eleonora Gambineri

**Affiliations:** ^1^Anna Meyer Children's Hospital, University of Florence, Florence, Italy; ^2^Division of Pediatric Oncology/Hematology, Meyer University Children's Hospital, Florence, Italy; ^3^Department of Neurosciences, Psychology, Drug Research and Child Health (NEUROFARBA), University of Florence, Florence, Italy

**Keywords:** immune dysregulation, primary immunodeficiencies, IPEX, regulatory T cells, CD25, STAT5B

## Abstract

CD25, Signal transducer and activator of transcription 5B (STAT5B) and Forkhead box P3 (FOXP3) are critical mediators of Interleukin-2 (IL-2) signaling pathway in regulatory T cells (Tregs). CD25 (i.e., IL-2 Receptor α) binds with high affinity to IL-2, activating STAT5B-mediated signaling that eventually results in transcription of FOXP3, a master regulator of Treg function. Consequently, loss-of-function mutations in these proteins give rise to Treg disorders (i.e., Tregopathies) that clinically result in multiorgan autoimmunity. Immunodysregulation, Polyendocrinopathy Enteropathy X-linked (IPEX), due to mutations in *FOXP3*, has historically been the prototype of Tregopathies. This review describes current knowledge about defects in *CD25, STAT5B*, and *FOXP3*, highlighting that these disorders both share a common biological background and display comparable clinical features. However, specific phenotypes are associated with each of these syndromes, while certain laboratory findings could be helpful tools for clinicians, in order to achieve a prompt genetic diagnosis. Current treatment strategies will be outlined, keeping an eye on gene editing, an interesting therapeutic perspective that could definitely change the natural history of these disorders.

## Introduction

Inborn errors of immunity include a growing number of genetic disorders not only characterized by an increased susceptibility to infections, but also leading to autoinflammation and autoimmunity (1). These features are particularly displayed by Primary Immune Regulatory Disorders (PIRDs). One of the main mechanisms behind immune dysregulation in PIRDs is an alteration of regulatory T cell (Treg) function: therefore, these particular types of PIRDs have been called Tregopathies (2). Immunodysregulation, Polyendocrinopathy Enteropathy X-linked (IPEX) syndrome has historically been a model of immune dysregulation and Tregopathy (3). Immunodysregulation, Polyendocrinopathy Enteropathy X-linked syndrome is due to a mutation in *FOXP3* (Forkhead Box P3, a master regulator of Treg function) and is characterized by a triad of intractable diarrhea, type 1 diabetes mellitus (T1DM), and eczema, as well as other autoimmune features (4). More recently, similar clinical phenotypes with a wild-type *FOXP3* have been identified and subsequently named “IPEX-like disorders” (5). The genetic defect behind these diseases may involve several immunological pathways, such as cell-contact dependent suppression (e.g., CTLA4 haploinsufficiency, LRBA deficiency) (6, 7), JAK-STAT signaling (e.g., STAT1 and STAT3 Gain-of-function) (8–11), or homeostasis, fitness, and maintenance of Treg cells (CD25 and STAT5B deficiencies) (5, 12).

In detail, both CD25 (i.e., IL-2 receptor α chain, IL-2Rα) and STAT5B (Signal Transducer and Activator of Transcription 5B) play a key role in the InterLeukin-2 (IL-2)-dependent regulation of FOXP3 expression (13, 14). Therefore, defects in the IL-2 signaling axis severely affect the function of Treg cells, which is strictly dependent on FOXP3 (15). Herein, we review current knowledge about defects in *CD25, STAT5B*, and *FOXP3* genes, from molecular insights to clinical presentations, highlighting the fact that defects in the same signaling pathway give rise to similar disease phenotypes. However, each of these disorders also displays peculiar clinical and immunological features, whose knowledge would be helpful in order to achieve an early diagnosis. Finally, we will describe current treatment options and future therapeutic perspectives for these emergent disorders of immune dysregulation.

## IL-2 Axis: Molecular Insights

**Figure 1:**
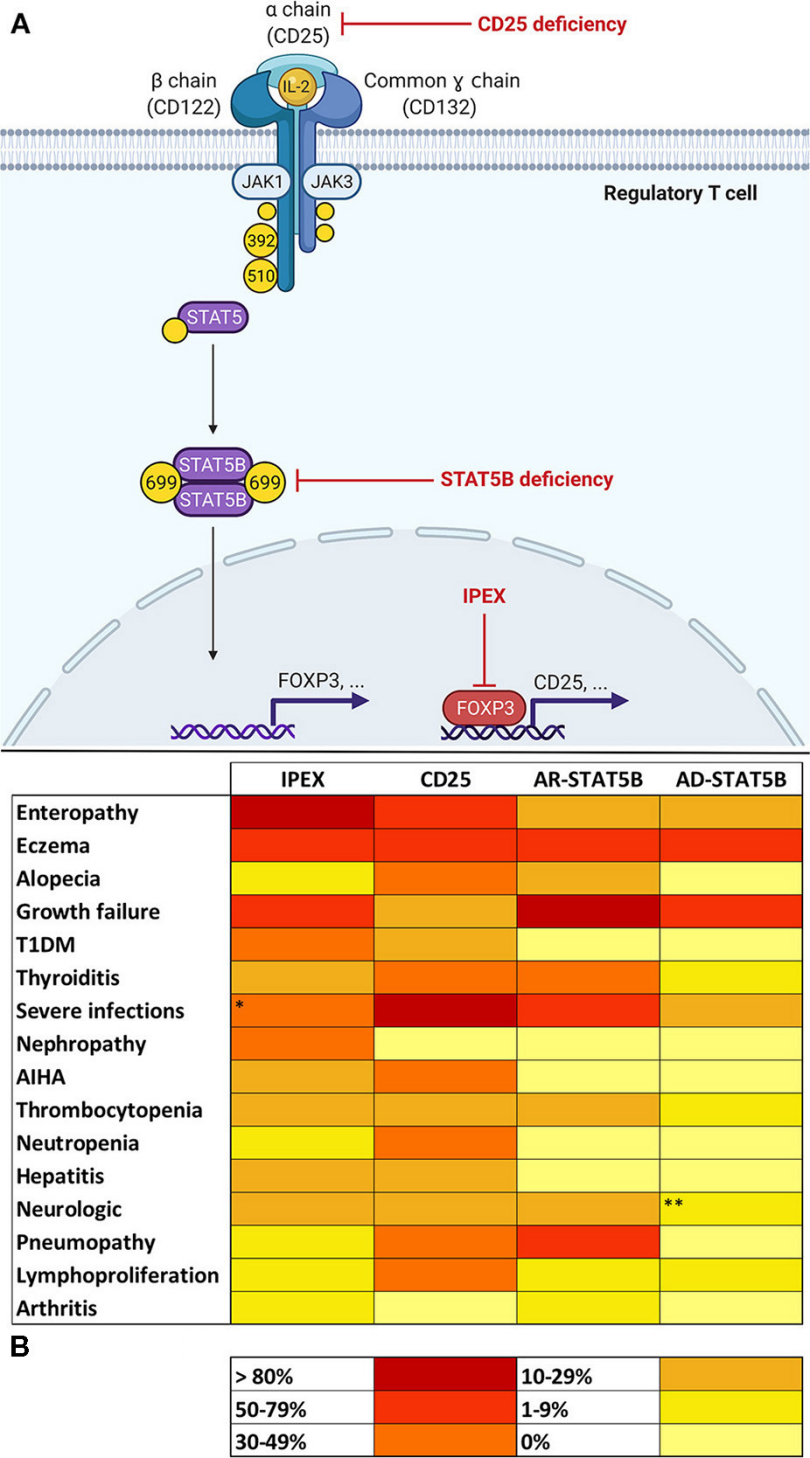
**(A)** Molecular basis of defects in the IL-2 signaling axis in Tregs. In regulatory T cells (Tregs), Interleukin-2 (IL-2) binds to a high-affinity trimeric receptor composed of an α (CD25), β (CD122), and common γ chain. The cytoplasmic domains of CD122 and γ chain are coupled to Janus kinases (JAKs) 1 and 3, respectively. Occupancy of IL-2 receptor (IL-2R) determines the activation of JAKs 1–3 and the subsequent phosphorylation of tyrosine residues on β and γ chains' cytoplasmic tails. In detail, the phosphorylation of tyrosines 392 and 510 on CD122 is paramount for the recruitment of signal transducers and activator of transcription 5 (STAT5). Activation of STAT5B in Tregs relies on JAK1-mediated phosphorylation of tyrosine 699. Phosphorylated STAT5B (pSTAT5B) is able to dimerize and migrate to the nucleus in order to bind DNA and induce transcription of several gene targets, including *FOXP3* (Forkhead Box P3). FOXP3 regulates expression of a variety of genes necessary for Treg function, including the up-regulation of CD25. This provides a positive feedback loop that allows—via STAT5 signaling—Treg maintenance and survival. Red arrows show the sites of molecular defects in CD25 and STAT5B deficiencies and in Immunodysregulation, Polyendocrinopathy Enteropathy X-linked (IPEX). **(B)** Heat map showing frequencies of clinical manifestations in IL-2 signaling axis defects. ^*^Data about severe infections refers to patients who did not yet start immunosuppressive drugs or did not yet undergo hematopoietic stem cell transplantation (32). ^**^Microcephaly reported in three patients belonging to the same family was not considered, since its association with STAT5B deficiency is uncertain (20). AR, autosomal recessive; AD, autosomal dominant.

Three distinct subunits compose the high affinity IL-2 receptor (IL-2R): α (CD25), β (CD122), and γ (i.e., common γ chain, CD132) (Figure 1A). While β and γ subunits may together form a functional receptor, CD25 is essential in order to acquire a high affinity for IL-2 (16). Moreover, CD25 exclusively binds IL-2 and its expression is limited to early stages of thymocyte development and to activated mature T lymphocytes. In particular, high levels of CD25 are constitutively expressed by CD4^+^CD25^+^FOXP3^+^ Treg cells. This confers to Tregs the ability to respond to low concentrations of IL-2, which is critical to maintain FOXP3 expression and—thereafter—cellular function and survival (17, 18).

In Tregs, the induction of FOXP3 expression determined by IL-2 occurs via a STAT5-dependent mechanism (14). IL-2 binding to its receptor leads to the recruitment of Janus Kinases (JAKs) 1 and 3, which, respectively, couple to β and γ subunits and phosphorylate tyrosine residues (Y) on their cytoplasmic tails. Specifically, the phosphorylation of Y392 and Y510 permits the recruitment of STAT5A and STAT5B, which are also phosphorylated by JAKs on specific tyrosine residues (13, 19) [e.g., STAT5B's Y699 phosphorylation appears critical for its activation (20)]. At this point, phosphorylated STAT5 (pSTAT5) molecules are able to dimerize and migrate to the Treg cell nucleus in order to bind DNA and regulate transcription of a variety of target genes including *FOXP3* (17).

STAT5A and STAT5B present peptide sequence similarities of >90% (21). As previously mentioned, STAT5B deficiency in humans leads to a specific disease phenotype (22). On the other hand, while in murine models STAT5A mutations lead to the loss of prolactin-dependent mammary gland development (23, 24), similar phenotypes have not been described in humans. This implies that—at least in humans—the roles of STAT5A and STAT5B are not fully redundant (25, 26). Moreover, STAT5 proteins also play a role in other cellular pathways, such as Granulocyte-Colony Stimulating Factor (G-CSF), Erythropoietin (EPO), and—most importantly—Growth Hormone (GH) signaling (25, 27, 28). In particular, GH interaction with its receptor activates signaling cascades that involve several STAT proteins. Among these, STAT5B appears to play a leading role in regulating Insulin-like Growth Factor (IGF-1) production (25).

The precise molecular mechanism of interaction between STAT5 and genes involved in Treg differentiation still needs to be elucidated. However, interaction with *FOXP3* seems unequivocal, since STAT5-binding motifs have been identified in the Conserved Non-coding DNA Sequence 2 (CNS2) of *FOXP3*'s promoter region (29, 30). High expression of FOXP3 induces the up-regulation of CD25, thus providing a positive feedback loop that allows—via STAT5 signaling—Treg maintenance and survival (17, 31). Indeed, FOXP3 is not necessary nor sufficient to display many aspects of the Treg phenotype, but its sustained expression is crucial for Treg stability, metabolic fitness and regulatory function (18).

Finally, IL-2 signaling influences several lymphocyte subsets and multiple mechanisms are still not fully understood (31). From this extremely simplified model of IL-2 axis (Figure 1A) we can deduce that CD25, STAT5B, and FOXP3 share a common biological background that may potentially justify the similar clinical phenotype shared by patients carrying defects in these genes.

## IPEX

Immunodysregulation, Polyendocrinopathy Enteropathy X-linked regopathies (32), being first described in 1982 (33) and genetically characterized in 2001 (34, 35). Immunodysregulation, Polyendocrinopathy Enteropathy X-linked syndrome is due to mutations in *FOXP3*, a syndrome is a PIRD that has historically gained the role of a prototype of monogenic autoimmune diseases and Tmaster transcription factor involved in the differentiation and function of Tregs (18). More than 300 mutations in *FOXP3* have been identified so far, both in coding and non-coding regions (36). However, precise genotype-phenotype correlations are lacking, reflecting the complex interplay between FOXP3 and other molecular components of the immune system, together with an emerging role of its epigenetic regulation (4).

### Clinical and Laboratory Features

Patients usually develop symptoms very early in infancy (Table 1) and intrauterine onset has been sporadically reported (37–39). The classical triad of clinical manifestations includes intractable diarrhea, type 1 diabetes mellitus and eczema. Diarrhea is a hallmark of IPEX, being reported in more than 90% of cases (32); however other gastrointestinal manifestations may develop [e.g., gastritis (40)]. Similarly, different endocrine (e.g., thyroiditis) and cutaneous findings (e.g., alopecia) are possible, as well as other organs may be the target of the ongoing autoimmune process. In detail, nephropathy, cytopenias, and hepatitis are not rare (41–43), while lymphoproliferation, severe food allergies, or other organs involvement are less frequently displayed (44–46). Moreover, severe infections are reported, but may be a consequence of immunosuppressive (IS) therapy rather than immune dysfunction itself (32). However, the disease course is somehow variable since several atypical cases have been recently described (47–50). Such reports expand the clinical spectrum of this disorder, highlighting the fact that IPEX could potentially be underdiagnosed (51).

**Table 1:** Clinical and laboratory features of IPEX.

**IPEX: Clinical features (32)**			**IPEX: Laboratory features (36)**		
	***N***	**Percentage (%)**		**Median**	**IQR**
Total patients	95	100	Total WBC × 1,000 (cell/μl)^†^	11.7	9.9–16.7
Enteropathy	85	89.5	Total lymphocytes × 1,000 (cell/μl)	2.4	1.3–3.2
Eczema	71	74.7	Total neutrophils × 1,000 (cell/μl)	2.2	0.2–4.9
Alopecia	8	8.4	Total eosinophils × 1,000 (cell/μl)^†^	1.1	0.5–3.1
Growth failure	71	74.7	Total T CD3+ × 1,000 (cell/ μl)	2.7	1.2–4.3
T1DM	42	44.2	Total T CD4+ × 1,000 (cell/ μl)	1.6	0.6–3.1
Thyroiditis	16	16.8	Total T CD8+ × 1,000 (cell/ μl)	0.7	0.4–1
Severe infections^*^	15/34^*^	44.0	Total CD19+ B cells × 1,000 (cell/μl)	0.6	0.1–0.8
Nephropathy	33	34.7	Total NK cells (cell/μl)	200	98–566
AIHA	26	27.4	Total IgG (mg/dl)	656	257–873
Thrombocytopenia	12	12.6	Total IgA (mg/dl)	29	8–94
Neutropenia	6	6.3	Total IgM (mg/dl)	52.6	32–182
Hepatitis	19	20.0	Total IgE (IU/ml)^†^	1,670	230–3,698
Neurologic	16	16.8	Positive autoantibodies (*N*, %)	153/272	71%
Food allergies	13	13.7			
Pneumopathy	9	9.5			
Lymphoproliferation	9	9.5			
Arthritis	8	8.4			
Pancreatitis	2	2.1			
Cardiac	2	2.1			
Ocular	2	2.1			
Months at onset (median, range)	2	0–11.3			

*Data are reported from a recent cohort (32) and review (36). N, number; IQR, interquartile range; T1DM, type 1 diabetes mellitus; AIHA, autoimmune hemolytic anemia; WBC, white blood cells; NK, natural killer*.

* *Data about severe infections refers to patients who did not yet start immunosuppressive drugs or did not yet undergo hematopoietic stem cell transplantation*.

† *Higher compared to healthy ranges for infants*.

Elevated IgE levels and hypereosinophilia are the most consistent laboratory abnormalities. Generally, other immunoglobulin levels are in range and lymphocyte subpopulations are conserved (36). Forkhead box P3 expression is not always abrogated and may be variable, according to the type of mutation; therefore, flow cytometry cannot be used as a screening test in IPEX (17). Moreover, FOXP3 expression is not correlated to disease severity (52). A wide range of autoantibodies has been reported, with anti-villin and anti-harmonin being particularly characteristic of IPEX (53, 54). However, genetic testing remains the gold standard for diagnosis and sequencing of both coding and non-coding regions of *FOXP3* must be assessed, in order to cover every potential site of known pathogenic mutations (5).

### Treatment Strategies

Therapies in IPEX are first of all supportive (parenteral nutrition, fluids, albumin, antibiotics), since clinical presentation is usually life-threatening and must include hormone-replacement therapies in case of endocrinopathy (e.g., insulin, levothyroxine) (4). Nevertheless, the two main treatment options are immunosuppression (IS) and allogeneic Hematopoietic Stem Cell Transplantation (HSCT). Most IPEX patients respond at least temporarily to the combination of steroids with either cyclosporine A or tacrolimus, however in recent years rapamycin proved to be the most beneficial IS drug (55, 56), though not influencing disease-free survival (32). Hematopoietic Stem Cell Transplantation is a potentially curative strategy, though bearing a high mortality rate in the first years following transplantation and depending on pre-HSCT clinical conditions (32). Recently, site-specific gene editing using CRISPR/Cas9 technology proved to be a promising therapeutic strategy, though only *in vitro* studies on *FOXP3*-mutated human cells have been performed (57). Future clinical developments of this approach may result in a potentially curative and non-invasive therapy for this disorder.

## CD25 Deficiency

CD25 (or IL-2Rα) deficiency is a very rare PIRD, with an autosomal recessive inheritance. Nine different types of causative mutations in nine patients have been described so far (58–64). The majority of mutations involve one of the two “sushi-like” domains (D1 and D2) of *IL-2R*α, which are in close contact with one another via an inter-domain sequence and several disulfide bonds (58, 65). Both point and frameshift mutations have been reported, however it is not yet clear whether the production of a dysfunctional or a truncated transcript influences disease phenotype.

### Clinical and Laboratory Features

The clinical tableau is dominated by both Severe Combined ImmunoDeficiency-like (SCID-like) features and overwhelming autoimmunity (17). These manifestations reflect the pleiotropic effects of IL-2, which plays a crucial role in the proliferation and development of T CD4^+^ lymphocyte subsets, in effector and memory T CD8^+^ activity and—most importantly—in Treg function (19).

Disease onset is strikingly early in life with a median age at onset of 1.25 months (Table 2). The clinical presentation may vary: severe Cytomegalovirus (CMV) or other types of infection can occur as presenting symptoms (59, 60, 66, 67), but also autoimmune manifestations may be the first sign of disease (58, 61). Interestingly, autoimmune features are somehow superimposable to those seen in IPEX, since all three components of the clinical triad (i.e., enteropathy, dermatitis, and T1DM) are reported. However, patients with CD25 deficiency display a more pronounced susceptibility to infections, which is reported in approximately 90% of cases. Severe viral infections are more frequently described, but also bacterial and fungal agents [e.g., esophageal candidiasis (59)] or recurrent infections (62) may seriously compromise the clinical conditions. Importantly, gastrointestinal infections (e.g., CMV enteritis) may further complicate the ongoing autoimmune enteropathy (61). Autoimmune cytopenias and lymphoproliferation are also described and may concomitantly occur (60, 63), potentially mimicking an Autoimmune lymphoproliferative syndrome-like (ALPS-like) clinical phenotype (68). Less frequent—though severe—autoimmune features are pneumopathy (64), hepatitis (63) and small-vessel pulmonary vasculitis with positive anti-GBM (Glomerular Basal Membrane) antibodies, as of Goodpasture's disease (63, 69).

**Table 2:** Clinical and laboratory features of CD25 deficiency.

**CD25 deficiency: Clinical features**			**CD25 deficiency: Laboratory features**		
	***N***	**Percentage (%)**		***N***	**Percentage**
**Total patients**	9	100	**Total patients**	9	100%
**Infections**	8	88.9	**IgG**	3 high	33.3% high
**Viral**	6	66.7	**IgA**	2 high, 1 low	22.2% high, 11.1% low
CMV	5	55.6	**IgM**	1 high	11.1% high
VZV	1	11.1	**IgE**	4 high	44.4% high
**Bacterial**	5	55.6	**Lymphopenia**	1	11.1%
**Fungal**	1	11.1	**Total CD3**	1 low	11.1% low
**Recurrent**	3	33.3	**Total CD4**	3 low	33.3% low
**Dermatitis**	7	77.8	**Total CD8**	2 high, 1 low	22.2% high, 11.1% low
Eczema	5	55.6	**Total CD19**	3 low	33.3% low
Alopecia	3	33.3	**Total CD56**	1 high, 1 low	11.1% high, 11.1% low
**Enteropathy**	6	66.7	**Auto-antibodies**	5	55.6%
**Endocrine**	4	44.4			
Thyroiditis	3	33.3			
T1DM	2	22.2			
**Lymphoproliferation**	3	33.3			
Lymphoadenopathy	3	33.3			
HSM	2	22.2			
**Cytopenias**	3	33.3			
AIHA	3	33.3			
Neutropenia	3	33.3			
Thrombocytopenia	2	22.2			
**Pneumopathy**	3	33.3			
**Growth failure**	2	22.2			
**Autoimmune Hepatitis**	1	11.1			
**Vasculitis**	1	11.1			
**Neurologic**	1	11.1			
**Months at onset (median, range)**	1.25	(0.2–7)			

*N, number; CMV, Cytomegalovirus; VZV, Varicella-Zoster virus; T1DM, type 1 diabetes mellitus; HSM, hepato-splenomegaly; AIHA, autoimmune hemolytic anemia*.

Laboratory tests show signs of immune dysregulation such as increased immunoglobulin levels (mildly elevated IgE, but also higher levels of IgG, IgA, and IgM have been described); nevertheless, low IgA and IgG4 concentrations have occasionally been reported (59, 64). Similarly, a low or inverted CD4^+^/CD8^+^ ratio seems recurrent, possibly explained by a better response to IL-2 by CD8^+^ T cells, which have a higher expression of CD122 (IL-2R β subunit) (2). All tested patients had an absence of CD25 on T cell surface, while a recent report identified intracytoplasmic CD25 expression in a patient carrying a homozygous missense mutation (58). Flow cytometry could therefore be used as a potential screening tool, in order to discriminate between IPEX and CD25 deficiency (58); however, gene sequencing is recommended to confirm diagnosis in any case (17).

### Treatment Strategies

Both antimicrobial and immunosuppressive therapy are needed to control severe infections and autoimmune manifestations, together with significant supportive care. Given the clinical severity and the early onset of disease presentation, HSCT appears to be the only curative possibility for these patients and was successfully performed in two cases (58, 59). Exogenous IL-2 therapy would theoretically be possible, in order to stimulate the low affinity IL-2 receptor β chain. Up to now, only *in vitro* studies in one affected patient showed a dose-dependent proliferation to recombinant human IL-2 (rhIL-2) (58), but future research may shed more light on this intriguing therapeutic perspective.

## STAT5B Deficiency

STAT5B deficiency gives rise to another form of PIRDs, typically associated with severe growth failure, highlighting its crucial role in the signaling pathways of IL-2, IL-15, and GH, as well as of other cytokines and growth factors (25). Fourteen different mutations in *STAT5B* (missense, non-sense, deletions, and frameshifts) have been identified so far (12, 26, 70–73). The majority of these involve the Coiled-coiled domain—which is important for protein–protein interactions and nuclear import (74)—, the DNA-binding and the Src homology 2 (SH2) domains, located right upstream of the essential Y699. Classically, STAT5B deficiency was described as an autosomal-recessive disorder (AR-STAT5B) and a total of 14 patients carrying homozygous mutations have been reported since 2003 (22). However, starting from 2017 (75), 13 cases of autosomal-dominant STAT5B deficiency (AD-STAT5B) have been described, displaying a variable disease penetrance. Of note, while germline loss-of-function mutations in *STAT5B* gene are associated with immune dysregulation, somatic gain-of-function mutations have been reported in distinct types of hematologic malignancies (76–78). In light of this, the prevalence of inactivating *STAT5B* mutations remains difficult to ascertain. While in front of the autosomal recessive form it is somehow mandatory to suspect a genetic disorder, AD-STAT5B shows a milder clinical presentation, and its incidence could possibly be underestimated. A better knowledge of the clinical and laboratory characteristics (described below and in Table 3)—may contribute to raise suspicion of these disorders, permitting to achieve an earlier diagnosis.

**Table 3:** Clinical and laboratory features of STAT5B deficiency.

**STAT5B deficiency: Clinical features**						**STAT5B deficiency: Laboratory features**					
	**AD-STAT5B**		**AR-STAT5B**				**AD-STATB**		**AR-STAT5B**		
	***N***	**Percentage (%)**	***N***	**Percentage (%)**	***p*-value**		***N***	**Percentage (%)**	***N***	**Percentage (%)**	***p*-value**
**Low stature**	9	69.2	14	100	**0.025**	**GH insensitivity**	6	46.2	14	100.0	**0.001**
**Latest Stature SDS** (median, range)	−2.84	(−0.4 to −5.3)	−5.81	(−4.7 to −9.9)	**<0.00001**	**High Prolactin**	3	23.1	5	35.7	0.6776
**Delayed bone age**	5	38.5	8	57.1	0.332	**Total IgG**	2 low	15.4	4 high	28.6	–
**Face deformities**	1	7.7	8	57.1	**0.0128**	**Total IgA**	n.d.	n.d.	4 high. 1 low	28.6% high 7.1% low	–
**Infections**	2	15.4	11	78.6	**0.0018**	**Total IgM**	1 high	7.7	2 high, 1 low	14.2% high, 7.1% low	–
**Viral infections**	0	0.0	5	35.7	**0.0407**	**Total IgE**	7 high	53.8	3 high	21.4	0.1201
VZV	0	0.0	5	35.7	**0.0407**	**Total Lymphocytes**	1 high	7.7	2 low	14.3	–
HSV	0	0.0	1	7.1	1	**Total T CD3**	n.d.	n.d.	3 low	21.4	–
**Bacterial infections**	2	15.4	6	42.9	0.2087	**Total T CD4**	n.d.	n.d.	5 low	35.7	–
**Fungal infections**	0	0.0	1	7.1	1	**Total T CD8**	n.d.	n.d.	5 low	35.7	–
**Recurrent infections**	2	15.4	10	71.4	**0.0063**	**T CD8 Effector Memory cells**	1 high	7.7	3 high	21.4	–
**Intestitial pneumopathy**	0	0.0	7	50.0	**0.0058**	**B memory switched**	n.d.	n.d.	3 high	21.4	–
**Enteropathy**	2	15.4	3	21.4	1	**Total NK cells**	n.d.	n.d.	2 low	14.3	–
**Dermatitis**	7	53.8	12	85.7	0.07	**Total Tregs**	n.d.	n.d.	6 low	42.9	–
**Eczema**	7	53.8	10	71.4	0.345	**Positive Autoantibodies**	2	15.4	7	50.0	–
**Alopecia**	0	0.0	2	14.3	0.4815	**TOTAL**	**13**	**100**	**14**	**100**	
**Thyroiditis**	1	7.7	6	42.9	0.0768						
**Lymphoproliferation**	1	7.7	1	7.1	1						
**Cytopenias**	1	7.7	2	14.3	1						
**Thrombocytopenia**	1	7.7	2	14.3	1						
**Arhtritis**	0	0.0	1	7.1	1						
Neurologic^**^	1^**^	7.7	2	14.3	1						
**TOTAL**	**13**	**100**	**14**	**100**							

*p-values were calculated with t-test, Pearson's chi-square test or Fisher's exact test according to the type of variable and sample size. A p-value of 0.05 or lower was considered statistically significant and written in bold. p-values were not calculated if data for one of the two disorders were missing or incomplete*.

** *Microcephaly reported in three patients belonging to the same family was not considered, since its association with STAT5B deficiency is uncertain (20). AD, autosomal-dominant; AR, autosomal-recessive; N, number; SDS, standard deviation score; VZV, Varicella-Zoster virus; HSV, Herpes Simplex virus; GH, growth hormone; NK, natural killer; Tregs, regulatory T cells; n.d., no data*.

### Autosomal-Recessive STAT5B Deficiency

Growth failure is the cardinal feature of AR-STAT5B and usually develops after birth, resulting in severe short stature (height ranging from −4.7 to −9.9 Standard Deviation Score, SDS). Delayed bone age and delayed puberty were also noted in some cases (12, 79–82). Typically, hormonal tests show a phenotype of GH insensitivity (i.e., normal levels of GH and GH-Binding Protein, low levels of IGF-1, IGFBP-3 and Acid Labile Subunit) (79), while prolactin levels may be elevated (83, 84). GH-therapy trials were undertaken in some cases but always proved to be ineffective (12, 79, 81, 83).

However, what undoubtedly differentiates AR-STAT5B from other GH-insensitivity syndromes (85, 86) is the combination of GH-resistant growth failure, infectious diathesis, and autoimmunity. Recurrent or severe viral and bacterial infections develop in the majority of cases and may further compromise respiratory conditions in patients displaying interstitial lung disease (80). Pneumopathy is the most severe autoimmune organ involvement, potentially leading to death (64). Other possible autoimmune manifestations include dermatitis (mainly eczema), thyroiditis, thrombocytopenia, arthritis, and enteropathy (17).

Since STAT5B is paramount in signaling of IL-2 and IL-15—which are key growth factors for T and NK cells respectively—, moderate CD4^+^, CD8^+^, and NK cell lymphopenia is frequently displayed, potentially explaining the increased susceptibility to viral infections (17). Importantly, Treg levels were found reduced in a significant proportion of patients. However, recent findings by Foley et al. showed that the percentage of CD4^+^CD25^+^CD127^low^ T cells reduces over time, suggesting that STAT5B deficiency impairs the preservation of FOXP3 expression, rather than the genesis of FOXP3^+^ Tregs. In parallel, an increase in CD8^+^ T-effector memory cells became significant over time, suggesting a role of STAT5B in proliferation and survival of such lymphocytes, though still not fully elucidated (72). Finally, B cell and immunoglobulin levels may be increased, with positive autoantibodies, which are consistent with the ongoing autoimmune manifestations (17).

### Autosomal-Dominant STAT5B Deficiency

Recent research unveiled specific heterozygous missense mutations in *STAT5B* that exert a dominant-negative activity and give rise to an AD-STAT5B phenotype (20, 73, 75). Mutant proteins showed to be phosphorylated upon stimulation, but either lost the ability to migrate toward the nucleus or failed to bind STAT5B response elements. However, these variants were able to dimerize with wild-type STAT5B, hampering its function and explaining the pathogenicity in heterozygous carriers (20).

Clinically, AD-STAT5B presents with a milder phenotype, if compared to the autosomal-recessive form (Table 3). Growth failure is significantly less severe and affected family members with normal stature have been reported (20), highlighting that prevalence of AD-STAT5B could be widely underestimated, due to an incomplete disease penetrance. Importantly, interstitial lung disease has never been reported in these patients and the only severe infections described were sporadic episodes of pneumonia. Eczema and elevated IgE levels seem to be recurring features, while microcephaly has been occasionally reported in three patients belonging to the same family (20). Notably, IGF-1 levels can be low or normal and few patients showed a partial response to GH or exogenous IGF-1 therapy, suggesting a phenotype of partial GH insensitivity.

### Treatment Strategies

In both AR and AD-STAT5B deficiencies treatment is usually supportive. As previously mentioned, GH replacement and IGF-1 trials resulted always ineffective in the recessive form, while partial benefit was seen in AD-STAT5B (20, 73). Antibiotic prophylaxis may be helpful in case of susceptibility to infections, while immune suppression resulted in controversial success for autoimmune manifestations. No therapies were significantly beneficial for the severe lung disease that affects AR-STAT5B patients, which led to lung transplantation in one case (83). Hematopoietic Stem Cell Transplantation has never been performed for this disorder, even though it could be speculated that it would repair the profound immune dysfunction. Nevertheless, HSCT would not cure the severe growth failure and such impact should be seriously considered before attempting it (87).

## Discussion

Interleukin-2 seems critical for the development and function of Tregs; therefore, an aberration in specific steps of signaling pathway results in three PIRDs involving the IL-2 axis: IPEX, CD25, and STAT5B deficiencies. Besides sharing a common molecular background, these disorders display comparable clinical features (Figure 1B). However, disease manifestations are not always consistent and clinical hallmarks of each disorder can be identified, such as enteropathy for IPEX, life-threatening infections in CD25 deficiency and severe growth failure for STAT5B deficiency. Nevertheless, such disease manifestations are not specific and only an association with other clinical features (e.g., classical triad in IPEX, pneumopathy in AR-STAT5B), may raise suspicion of a particular genotype of IL-2 axis impairment. Multiorgan autoimmunity in IPEX is notably due to a dysfunction of thymus derived Tregs (4). On the other hand, the pronounced susceptibility to infections in CD25 deficiency is explainable by the multiple consequences of a defect in IL-2Rα, which both hampers Treg functionality and jeopardizes many aspects of adaptive immune response, including naïve CD8^+^ T cells' differentiation and function (19). A propensity to develop viral infections—however—is not so evident in IPEX and STAT5B deficiencies. We may speculate that compensating cytoplasmic and nuclear pathways downstream of IL-2R may counterbalance the lack of STAT5B and FOXP3 in STAT5B deficiency and IPEX, thereby assuring a satisfactory function of CD8^+^ T cells. In parallel, the pleiotropic role of STAT5B, which regulates signaling of GH and other growth factors, makes low stature a prerogative of STAT5B deficiency (25). Curiously, eczema is commonly observed throughout these disorders, consistently with the fact that skin findings are often a window into primary immunodeficiencies (88).

In addition to clinical features, laboratory findings may strengthen the suspect of one disease rather than another. Markedly elevated IgE and hypereosinophilia are frequent in IPEX, while a hormonal pattern of GH insensitivity is suggestive of STAT5B deficiency. *In vitro* evaluation of STAT5 phosphorylation in lymphocytes can help to reveal an altered pathway (89). Moreover, flow cytometry is a suitable tool in order to exclude or furtherly support the suspect of CD25 deficiency, since in this disorder Tregs have a typical CD25^−^FOXP3^+^ phenotype (58, 60, 61). In contrast, a FOXP3^low^ pattern or a decreased expression of FOXP3 over time is described in AR-STAT5B (72). These findings are consistent with current models of IL-2 signaling since FOXP3 maintenance seems to be dependent on STAT5B (19). Conversely, mutations in *CD25* are not sufficient to disrupt FOXP3 expression, possibly because of a residual activity performed by the β and γ subunits of IL-2 receptor. Finally, FOXP3 expression is not diagnostic in IPEX, since it may be abrogated or increased, depending on the type of mutation or IS treatment (4).

Gene sequencing remains the gold standard for diagnosis and may reveal pathogenic mutations even when in front of atypical or incomplete phenotypes, especially in IPEX (51). The increasing use of next-generation sequencing techniques could furtherly reveal genetic causes underlying milder phenotypes of immune dysregulation. For instance, patients with complete or partial GH insensitivity who also display eczema and/or elevated IgE, could be analyzed for AD-STAT5B. Indeed, the prevalence of heterozygous mutations in *STAT5B* is probably underestimated, since such phenotype has been recently discovered (75) and dominant-negative variants are already known for other STAT genes (90, 91). Importantly, truncating mutations have never been reported for AD-STAT5B, since they are unable to fulfill a dominant-negative action, which can only be achieved by the interaction between missense transcript and wild-type protein (20).

In conclusion, PIRDs involving the IL-2 axis have a similar immunopathogenesis and share common clinical features, though specific phenotypes are associated with each of these disorders. Flow cytometry and specific laboratory tests are helpful diagnostic tools, but only genetic testing can precisely identify these syndromes. Treatment is mainly supportive for STAT5B deficiency and HSCT can be curative in IPEX and CD25 deficiency, while gene-editing will hopefully be a definitive therapeutic option in the future.

## Author Contributions

EG and FC reviewed literature and performed analysis of data. FC wrote the original draft of the article. CF and EG supervised the work. All authors contributed to the article and approved the submitted version.

## Conflict of Interest

The authors declare that the research was conducted in the absence of any commercial or financial relationships that could be construed as a potential conflict of interest.
